# Caspofungin Weight-Based Dosing Supported by a Population Pharmacokinetic Model in Critically Ill Patients

**DOI:** 10.1128/AAC.00905-20

**Published:** 2020-08-20

**Authors:** Anne-Grete Märtson, Kim C. M. van der Elst, Anette Veringa, Jan G. Zijlstra, Albertus Beishuizen, Tjip S. van der Werf, Jos G. W. Kosterink, Michael Neely, Jan-Willem Alffenaar

**Affiliations:** a Department of Clinical Pharmacy and Pharmacology, University of Groningen, University Medical Center Groningen, Groningen, The Netherlands; b Department of Clinical Pharmacy, University Medical Center Utrecht, University Utrecht, Utrecht, The Netherlands; c Department of Critical Care, University of Groningen, University Medical Center Groningen, Groningen, The Netherlands; d Medisch Spectrum Twente, Intensive Care Center, Enschede, The Netherlands; e Department of Pulmonary Diseases and Tuberculosis, University of Groningen, University Medical Center Groningen, Groningen, The Netherlands; f Department of Internal Medicine, University of Groningen, University Medical Center Groningen, Groningen, The Netherlands; g Groningen Research Institute for Pharmacy, PharmacoTherapy, Epidemiology & Economy, University of Groningen, Groningen, The Netherlands; h Laboratory of Applied Pharmacokinetics and Bioinformatics, Children's Hospital of Los Angeles, Los Angeles, California, USA; i Sydney Pharmacy School, The University of Sydney, Sydney, New South Wales, Australia; j Westmead Hospital, Sydney, New South Wales, Australia; k Marie Bashir Institute of Infectious Diseases and Biosecurity, The University of Sydney, Sydney, New South Wales, Australia

**Keywords:** caspofungin, pharmacodynamics, pharmacokinetics, population pharmacokinetics, weight-based dosing

## Abstract

The objective of this study was to develop a population pharmacokinetic model and to determine a dosing regimen for caspofungin in critically ill patients. Nine blood samples were drawn per dosing occasion. Fifteen patients with (suspected) invasive candidiasis had one dosing occasion and five had two dosing occasions, measured on day 3 (±1) of treatment. Pmetrics was used for population pharmacokinetic modeling and probability of target attainment (PTA). A target 24-h area under the concentration-time curve (AUC) value of 98 mg·h/liter was used as an efficacy parameter. Secondarily, the AUC/MIC targets of 450, 865, and 1,185 were used to calculate PTAs for Candida glabrata, C. albicans, and C. parapsilosis, respectively. The final 2-compartment model included weight as a covariate on volume of distribution (*V*). The mean *V* of the central compartment was 7.71 (standard deviation [SD], 2.70) liters/kg of body weight, the mean elimination constant (*K_e_*) was 0.09 (SD, 0.04) h^−1^, the rate constant for the caspofungin distribution from the central to the peripheral compartment was 0.44 (SD, 0.39) h^−1^, and the rate constant for the caspofungin distribution from the peripheral to the central compartment was 0.46 (SD, 0.35) h^−1^. A loading dose of 2 mg/kg on the first day, followed by 1.25 mg/kg as a maintenance dose, was chosen. With this dose, 98% of the patients were expected to reach the AUC target on the first day and 100% of the patients on the third day. The registered caspofungin dose might not be suitable for critically ill patients who were all overweight (≥120 kg), over 80% of median weight (78 kg), and around 25% of lower weight (≤50 kg). A weight-based dose regimen might be appropriate for achieving adequate exposure of caspofungin in intensive care unit patients.

## Abstract (precis)

The objective of this study was to develop a population pharmacokinetic model and to determine a dosing regimen for caspofungin in critically ill patients. Nine blood samples were drawn per dosing occasion. Fifteen patients with (suspected) invasive candidiasis had one dosing occasion and five had two dosing occasions, measured on day 3 (±1) of treatment. Pmetrics was used for population pharmacokinetic modeling and probability of target attainment (PTA). A target 24-h area under the concentration-time curve (AUC) value of 98 mg·h/liter was used as an efficacy parameter.

## TEXT

Caspofungin, an echinocandin antifungal drug, is used for the treatment of invasive candidiasis (1–3). The European Society of Intensive Care Medicine (ESICM) and the European Society of Microbiology and Infectious Disease (ESCMID) established a task force on the practical management of invasive candidiasis in critically ill patients (1). The expert panel of these combined societies recommended echinocandins as the primary therapy in critically ill patients with invasive candidiasis complicated by septic shock and multiorgan failure (1). Other guidelines also recommend echinocandins as a first-line treatment in critically ill patients (2, 4).

Previous studies have shown that caspofungin has both high pharmacokinetic variability and considerable risk of low exposure in critically ill patients (5–7). Currently, the caspofungin summary of product characteristics (SmPC) recommends a maintenance dose of 70 mg daily for patients weighing over 80 kg and a reduced dose for patients with lower body weight and for patients with moderate hepatic impairment (8). It has been suggested that the caspofungin dose should be escalated in critically ill patients to achieve adequate exposure (9, 10). Moreover, some studies have shown that patients with hepatic impairment might not require initial dose reduction, as after dosage alteration, lower exposure has been observed (10, 11).

The first objective of this study was to develop and validate a population pharmacokinetic model for caspofungin. The secondary objective was to determine a dosage regimen of caspofungin for critically ill patients.

## RESULTS

### Study population.

This study included 20 intensive care unit (ICU) patients. For five patients, the exposure was measured on two occasions (for two different dose regimens) and for 15 patients on one occasion, resulting in 219 caspofungin concentrations. Due to unforeseeable circumstances in the ICU care during the original study, six samples could not be obtained; however, each of these six samples were on different dosing occasions. The median age was 56 (minimum-maximum [min-max] range, 25 to 83) years, and the median weight was 78 (range, 48 to 139) kg. Two patients had severe liver damage with a Child-Pugh score of C. The patient characteristics and pharmacokinetic exposure analysis are described in Table 1.

**TABLE 1:** Patient characteristics^*a*^

Characteristic^*b*^	Value (*n* = 20; % or min-max range)
Male (*n*)	11 (55)
Median age, yr	56 (25–83)
Median wt, kg	78 (48–139)
Coadministration of prednisolone-hydrocortisone	11 (55)
CVVH	8 (40)
Median SAPS 3 score	59 (31–104)
Median serum albumin (g/liter)	20 (14–28)
Median CRP (mg/liter)	124 (56–287)
Median serum creatinine (mg/liter)	83 (40–466)
Median ALAT (u/liter)	35.5 (7–598)
Median ASAT (u/liter)	39 (12–1776)
Median ALP (u/liter)	122 (56–460)
Median GGT (u/liter)	85.5 (16–941)
Median bilirubin (mmol/liter)	7.5 (3–376)

a This table has been reproduced from reference 6.

b CVVH, continuous venovenous hemofiltration; CRP, C-reactive protein; ALP, alkaline phosphatase; GGT, gamma-glutamyltransferase.

### Population pharmacokinetic model.

During the modeling, one- and two-compartment pharmacokinetic models were tested. After stepwise linear regression analysis, albumin, sex, simplified acute physiology score (SAPS 3), bilirubin, ASAT (aspartate transaminase), ALAT (alanine transaminase), hemodialysis, and age were included as covariates in the model on different pharmacokinetic parameters. Overall, 24 models with different sets of covariates and error models were tested. All the tested models are described in Table S1 in the supplemental material.

The final model was a two-compartment model with normalized population median weight as a covariate on volume of distribution (*V*) using a gamma error model (*V* = *V*_0_ · weight/78). The final run gamma value was 0.654, which confirms that no significant noise was specified in the model (12). The mean *V* of the central compartment was 7.71 (standard deviation [SD], 2.70) liters/kg, the mean elimination rate constant (*K_e_*) was 0.09 (SD, 0.04) h^−1^, the rate constant for the caspofungin distribution from the central to the peripheral compartment was 0.44 (SD, 0.39) h^−1^, and the rate constant for the caspofungin distribution from the peripheral to the central compartment was 0.46 (SD, 0.35) h^−1^. The population median weight was included as a covariate in the final model, as it resulted in an improved goodness of fit and decreases in Aikake information criterion (AIC) and −2 log likelihood values.

**FIG 1:**
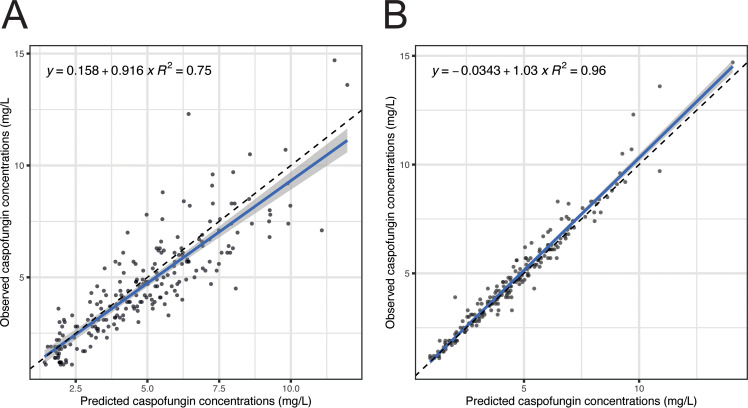
Goodness-of-fit plots for caspofungin. (A) Observed versus predicted population caspofungin concentrations. (B) Observed versus predicted individual caspofungin concentrations.

**FIG 2:**
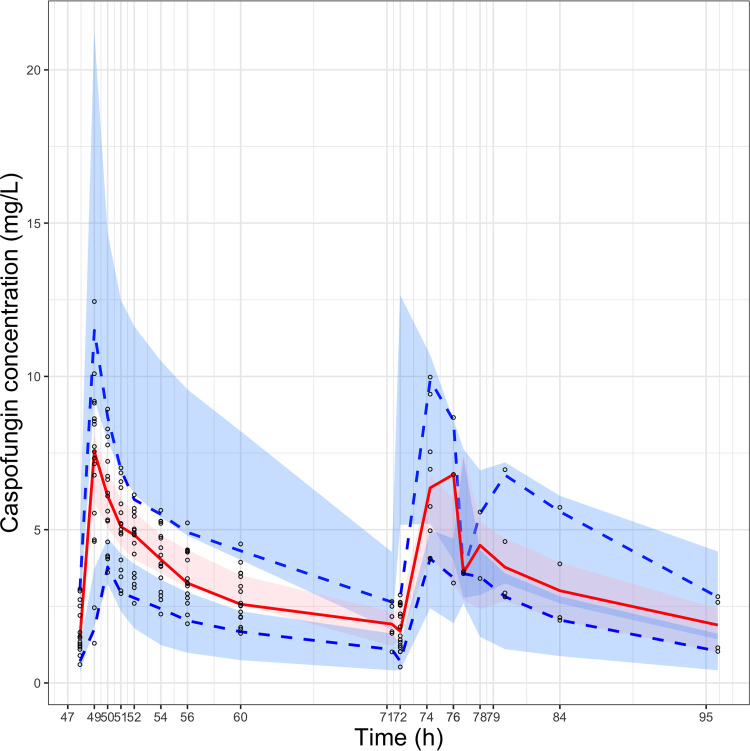
Prediction-corrected visual predictive checks (pcVPCs) for the final two-compartment pharmacokinetic model. The red line represents the median, and the blue dashed lines represent the 5th and 95th percentiles for the observed data.

**FIG 3:**
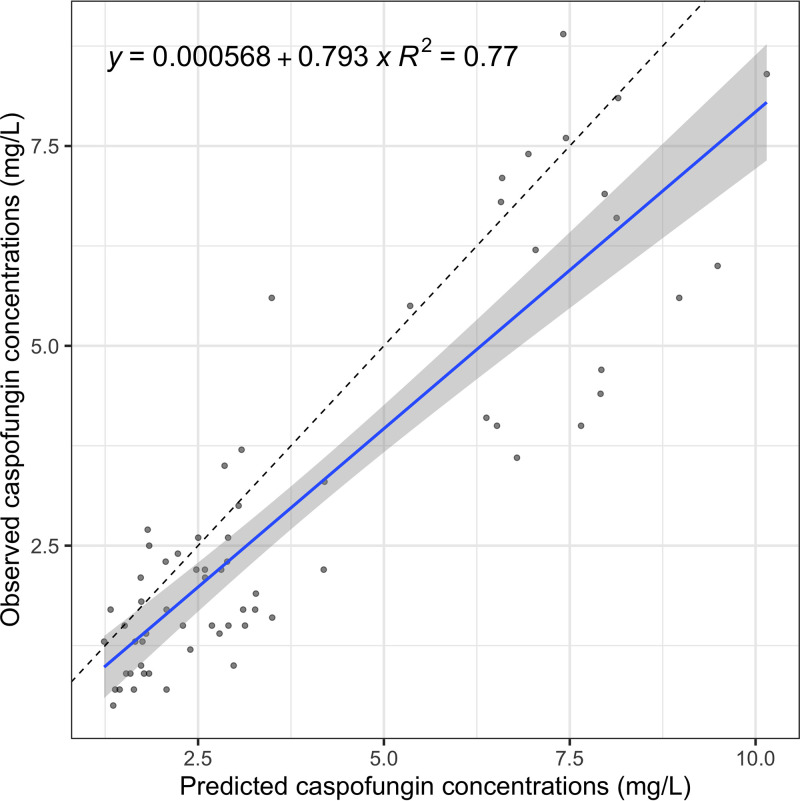
External validation with an independent cohort.

The final model population fit resulted in *r* of 0.75 and individual fit in *r *of 0.96. The goodness-of-fit plots for population and individual caspofungin concentrations are presented in Fig. 1. The final parameter estimates for the two-compartment population model are presented in Table 2. The visual predictive check showed good performance of the final model and did not reveal significant deviations or outliers. The visual predictive check plot is presented in Fig. 2. The external validation with the digitized data from Kurland et al. showed a fit of *r = *0.77 (Fig. 3), and data from Muilwijk et al. showed a fit of *r *= 0.83 (Fig. S1) (7, 13). The normalized prediction distribution error (NPDE) plots are presented in Fig. S3.

**TABLE 2:** Final parameter estimates for the two-compartment caspofungin population pharmacokinetic model

Pharmacokinetic parameter^*a*^	Mean	SD	Median	CV%
*K_e_* (h^−1^)	0.09	0.04	0.08	42.38
*V*_0_ (liters/kg)	7.71	2.70	7.20	34.98
*k*_cp_ (h^−1^)	0.44	0.38	0.28	88.02
*k*_pc_ (h^−1^)	0.46	0.35	0.34	75.98

a *K_e_*, elimination rate constant; *V*_0_, volume of distribution; *k*_cp_, rate constant for the caspofungin distribution from the central to the peripheral compartment; *k*_pc_, rate constant for the caspofungin distribution from the peripheral to the central compartment.

### Probability of target attainment (PTA).

To evaluate the caspofungin registered dose reported in the SmPC, fixed-dose regimens were simulated, where the population weight was centered around three weight bands: 50 kg, 78 kg (population median), and 120 kg. The 70-mg dose resulted in 73% of ∼50-kg patients, 14% of ∼78-kg patients, and 0% of ∼120-kg patients reaching the target area under the concentration-time curve (AUC; ≥98 mg·h/liter) for the first day of therapy. Other fixed-dose regimens are presented in Table 3.

**TABLE 3:** Probability of target attainment using fixed caspofungin dosing regimens in different weight categories

Loading dose	Maintenance dose	PTA (%) by weight category and AUC (mg·h/liter)											
		0–24 h						48–72 h					
		50 kg		78 kg		120 kg		50 kg		78 kg		120 kg	
		≥98	≥200	≥98	≥200	≥98	≥200	≥98	≥200	≥98	≥200	≥98	≥200
70 mg	50 mg	73	2	14	0	0	0	79	2	19	0	0	0
100 mg	70 mg	98	22	57	0	10	0	99	23	61	0	12	0
70 mg		73	2	14	0	0	0	98	15	53	0	11	0
100 mg		98	22	57	0	10	0	100	56	98	14	37	0

For the weight-based dosing regimen, a dose of 2 mg/kg on the first day (loading dose), followed by 1.25 mg/kg as a maintenance dose was the regimen that had the highest success rate. With this dose, 98% of the patients are expected to reach the target AUC (≥98 mg·h/liter) on the first day and 100% of the patients on the third day. This dose regimen exceeded the upper-threshold AUC of ≥200 mg·h/liter for 21% and 15% of the patients on the first and third day, respectively. All the weight-based dosages up to day 14 are presented in Table 4.

**TABLE 4:** Probability of target attainment using weight-based dosing regimens of caspofungin

Loading dose	Maintenance dose	PTA (%) by AUC (mg·h/liter)											
		0–24 h		48–72 h		120–144 h		192–216 h		264–288 h		312–336 h	
		≥98	≥200	≥98	≥200	≥98	≥200	≥98	≥200	≥98	≥200	≥98	≥200
2 mg/kg	1 mg/kg	98	21	91	6	88	5	89	5	89	5	89	5
1.5 mg/kg	1.25 mg/kg	83	3	99	13	100	16	100	17	100	18	100	18
2 mg/kg	1.25 mg/kg	98	21	100	15	100	16	100	17	100	18	100	18
1 mg/kg		22	0	77	2	88	5	89	5	89	5	89	5
1.5 mg/kg		83	3	100	25	100	31	100	33	100	33	100	33

**FIG 4:**
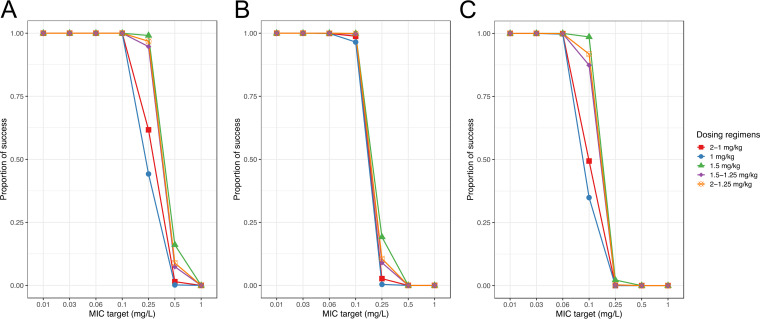
(A) Probability of target attainment on third day of therapy for AUC/MIC target of 450 for C. glabrata. (B) Probability of target attainment on third day of therapy for AUC/MIC target of 865 for C. albicans. (C) Probability of target attainment on third day of therapy for AUC/MIC target of 1,185 for C. parapsilosis.

The MIC/AUC targets for C. glabrata, C. albicans, and C. parapsilosis were analyzed with fixed and weight-based dose regimens. Using a MIC of 0.06 mg/liter from EUCAST clinical breakpoints for fungi, all of the weight-based and fixed-dose regimens reached the pharmacokinetic/pharmacodynamic (PK/PD) target at the third day for C. glabrata and C. albicans (14). However, for C. parapsilosis, using MICs of 0.25 mg/liter and 1 mg/liter, our proposed weight-based regimens were not appropriate (15). The fixed-dose regimens had an overall lower target attainment than weight-based regimens. All the PTAs with the MIC range from 0.01 to 1.0 mg/liter are presented in Table 5. The C. glabrata, C. albicans, and C. parapsilosis PTAs for the third day of therapy with different weight-based dose regimens are presented in Fig. 4A to C.

**TABLE 5:** Probability of target attainment for AUC/MIC targets of 450, 865, and 1,185 for 3rd day of caspofungin therapy (48 to 72 h)

Dose (mg/kg) and species	PTA (%) for MIC (mg/liter) of^*a*^:						
	0.01	0.03	0.06	0.1	0.25	0.5	1.0
2–1 mg/kg							
C. glabrata	100	100	100	100	62	2	0
C. albicans	100	100	100	99	3	0	0
C. parapsilosis	100	100	100	49	0	0	0
1.5–1.25 mg/kg							
C. glabrata	100	100	100	100	95	7	0
C. albicans	100	100	100	100	9	0	0
C. parapsilosis	100	100	100	87	0	0	0
2–1.25 mg/kg							
C. glabrata	100	100	100	100	97	9	0
C. albicans	100	100	100	100	11	0	0
C. parapsilosis	100	100	100	92	0	0	0
1 mg/kg							
C. glabrata	100	100	100	100	44	0	0
C. albicans	100	100	100	97	1	0	0
C. parapsilosis	100	100	98	35	0	0	0
1.5 mg/kg							
C. glabrata	100	100	100	100	99	16	0
C. albicans	100	100	100	100	19	0	0
C. parapsilosis	100	100	100	99	2	0	0
70 mg							
C. glabrata	100	100	100	100	29	0	0
C. albicans	100	100	100	75	0	0	0
C. parapsilosis	100	100	98	26	0	0	0
70–50 mg							
C. glabrata	100	100	100	99	8	0	0
C. albicans	100	100	98	26	0	0	0
C. parapsilosis	100	100	48	6	0	0	0
100 mg							
C. glabrata	100	100	100	100	93	6	0
C. albicans	100	100	100	99	9	0	0
C. parapsilosis	100	100	100	84	0	0	0
100–70 mg							
C. glabrata	100	100	100	100	31	0	0
C. albicans	100	100	100	82	0	0	0
C. parapsilosis	100	100	98	28	0	0	0

a An AUC/MIC target of 450 was used for Candida glabrata, 865 for Candida albicans, and 1,185 for Candida parapsilosis.

## DISCUSSION

We present a caspofungin population pharmacokinetic model developed using Pmetrics. In Pmetrics, the nonparametric adaptive grid and parametric iterative two-stage Bayesian approaches provide a robust pharmacokinetic model that is able to capture subgroups and outliers in the population (16). Caspofungin population pharmacokinetics were best described using a two-compartment pharmacokinetic model using population median weight as a covariate on volume of distribution (*V*). This is in agreement with a previous caspofungin model using nonlinear mixed-effects modeling (NONMEM); however, in that model, plasma protein concentration was also included as a covariate (9).

It has been shown in healthy adults that with increasing weight, both *V* and clearance (CL) increase (17). In addition, a study conducted in critically ill patients reported a *V* of 7.03 liters and CL of 0.54 liters/h, which is similar to our findings; however, with a *K_e_* of 0.09, our CL is approximately 0.7 liters (7). Other models have also included weight as a covariate and obtained similar results (10, 18). Furthermore, Nguyen et al. described that caspofungin exposure was influenced by albumin concentration and body weight (19). During our model development, albumin was also tested as a covariate; however, this did not improve our final model, which might be because albumin was not as frequently measured.

This study suggests that the registered caspofungin dose is not sufficient to achieve PTA for all overweight individuals (≥120 kg) and over 80% of average-weight and around 25% of lower-weight (<50 kg) critically ill patients. Our previous analysis suggested using a weight-based dosage regimen of 1 mg/kg once daily; however, probability of target attainment was not addressed (6, 20). The current dosing regimen is based on a validated nonparametric population pharmacokinetic model and subsequent Monte Carlo simulations. Using this method, we could calculate the PTAs for different dosing regimens based on the developed population model. The most appropriate dosage regimen reaching a 24-h steady-state AUC value of 98 mg·h/liter for over 95% of simulated patients was a 2-mg/kg loading dose followed by a 1.25-mg/kg daily dose. This approach might result in overall higher daily dosing than that with fixed dosing; however, toxicity is not a major concern with caspofungin. A study with doses of up to 200 mg daily for an extended period of time showed good tolerability was observed, with no described dose-limiting toxicity (21). Additionally, a loading dose has been shown to be necessary to achieve the AUC target on day 1 (22). We are looking forward to the results of an ongoing prospective study investigating the impact of a caspofungin loading dose of 140 mg (https://clinicaltrials.gov/ct2/show/NCT02413892).

We calculated the PTAs for AUC/MIC targets that have been proposed in a murine study (24) and have also been implemented in multiple clinical studies (9, 10, 25). The AUC/MIC target of a MIC of 0.06 mg/liter was reached for all weight-based dosing regimens. However, as described previously, with the potentially increasing breakpoints and higher MIC targets for C. parapsilosis, the optimal dose may be even higher than that of our proposed weight-based dosing regimen to reach the proposed target (9). The latest EUCAST clinical breakpoints for fungi suggest that isolates that are susceptible to anidulafungin and micafungin should be considered susceptible to caspofungin, as there is significant variability between laboratories in reported MIC ranges (14). Martial et al. showed that, using the registered dosing regimen of caspofungin, the AUC/MIC target of 865 is not reached and a 100-mg loading dose may be appropriate for *Candida* species with a MIC of >0.125 mg/liter (10). Pérez-Pitarch et al. suggested fixed dosing regimens up to 200 mg daily to cover *Candida* species with increasing MIC (up to 0.25 mg/liter) (9). Furthermore, in most cases, at the start of the treatment, the MIC of the *Candida* species is not known. To avoid a delay in appropriate antifungal therapy, it is necessary to acquire adequate exposure to cover the susceptible *Candida* species.

Our population is not representative for patients with liver failure, since only two of the patients had severe liver failure (Child-Pugh score C). However, the population fit did not show significant discrepancies for these 2 patients. Furthermore, liver function markers aspartate aminotransferase (AST), alanine aminotransferase (ALT), and gamma-glutamyl transpeptidase (GGT) were not included as covariates in the final model, as these did not improve the population goodness of fit and other model parameters. Thus, these patients did not seem to form a different subgroup from the rest of the population. Caspofungin clearance seems not to have changed in the patients with Child-Pugh B and C, which explains why lower exposure was observed when doses were reduced (10, 11).

This study has some limitations. First, we did not take plasma protein binding into account while modeling, as we measured total caspofungin concentrations. As caspofungin is highly protein bound (∼97%), the extent of protein binding can change in ICU patients, and the measurement of unbound fractions may be useful; however, drug assessment can be difficult, as small absolute errors translate into large relative errors in highly protein-bound drugs (26, 27). Second, the PK/PD target for AUC is not well established in clinical trials, and the currently used targets are based on murine models only. These targets should be evaluated in prospective patient cohorts with clear outcome measures. With respect to this, we suggest guiding therapy with therapeutic drug monitoring to reach the optimal targets, as was performed in our initial study (6) and other studies (5, 28).

In summary, we developed a two-compartment nonparametric population pharmacokinetic model and designed PTAs using AUC and AUC/MIC as targets. A weight-based dose regimen of a 2-mg/kg loading dose and 1.25-mg/kg daily dose might be more appropriate for achieving adequate exposure of caspofungin in ICU patients than the standard fixed-dose regimen. This dosing regimen should be prospectively evaluated.

## MATERIALS AND METHODS

### Study population and sampling.

This study included data from a prospective study in 20 adult critically ill patients admitted to an ICU with suspected invasive candidiasis and treated with caspofungin (6). For more details about the study population, see our previous publication (6).

All patients received a loading dose of 70 mg on the first day of treatment. The subsequent dose was 50 mg for patients weighing ≤80 kg, 70 mg for patients weighing >80 kg, and 35 mg and 50 mg, respectively, for patients with moderate hepatic impairment (Child-Pugh score of 7 to 9) (8). Caspofungin was administered as a 1-h infusion.

As the steady state for caspofungin is reached on the second day after the loading dose, blood sampling was performed on day 3 (range, 2 to 4) (22). If the dose was changed due to an area under the concentration-time curve (AUC) value of <98 mg·h/liter, the sampling was repeated after 3 days. This AUC exposure has been shown to be achieved in healthy volunteers after standard dosing, and 1-log kill of C. albicans at an AUC of 98 mg·h/liter should be sufficient according to *in vivo* analysis (22, 24, 29). The rationale for this target is described in detail in our previous publication (6). The sampling was performed before the administration and 1, 2, 3, 4, 6, 8, 12, and 24 h after the start of the caspofungin infusion. Caspofungin plasma concentrations were measured using a validated liquid chromatography-tandem mass spectrometry assay (30).

### Population pharmacokinetic modeling.

The pharmacokinetic modeling, probability of target attainment, and visual predictive checks were performed using the nonparametric adaptive grid program (NPAG) in Pmetrics (version 1.5.2) for R (version 3.6.1) (Laboratory of Applied Pharmacokinetics and Bioinformatics, Los Angeles, CA) (16).

The covariate selection was performed using the PMstep command of Pmetrics. Each covariate was tested in a linear regression analysis on pharmacokinetic parameters to see if there was a significant effect on AIC value (*P* < 0.05). The covariates were retained in the model when the −2 log likelihood, AIC, and Bayesian information criterion (BIC) values improved significantly and/or resulted in an improved goodness-of-fit plot. The covariates, tested with a forward addition method, were weight, age, gender, concomitant administration of prednisolone/hydrocortisone, dialysis, aspartate aminotransferase (AST), alanine aminotransferase (ALT), gamma-glutamyl transpeptidase (GGT), bilirubin, albumin, C-reactive protein, leukocyte count, and simplified acute physiology score (SAPS 3).

### Model diagnostics.

The models were analyzed and compared using individual and population observed versus predicted goodness-of-fit plots, AIC, BIC, and −2 log likelihood. The prediction error was evaluated using bias (mean weighted prediction error) and imprecision (bias-adjusted mean weighted squared prediction error) for both the individual and population models. During the population modeling assay, error (standard deviation) and environmental noise were considered. For this, we used error polynomials in the following equation: standard deviation = *C*_0_ + *C*_1_ × observed concentration. The value 0.05 was used for *C*_0_ and 0.08 for *C*_1._ Gamma multiplicative and lambda additive error models were tested to estimate residual error (12, 31).

The prediction- and variability-corrected visual predictive checks (pcVPCs) were done to evaluate the performance of the final population pharmacokinetic model (32). The model was validated with two external digitized data sets from caspofungin pharmacokinetic studies on critically ill patients (7, 13). The data were digitized using WebPlotDigitizer (https://automeris.io/WebPlotDigitizer/), and a uniform distribution was used to sample random numbers from the weight range reported in the publication.

### Probability of target attainment.

The final population model was used for the Monte Carlo simulations (*n* = 1,000) to calculate the PTAs for different dosage regimens. For the primary PTA target, the 24-h steady-state AUC value of 98 mg·h/liter was used as an efficacy parameter and an AUC value of 200 mg·h/liter as an arbitrarily assigned upper threshold of twice the proposed efficacy target (22, 24, 29). The PTAs were simulated for fixed and weight-based dosing for day 1, 3, 6, 9, 12, and 14 of therapy. Fixed dosage regimens were a 70-mg loading dose on day 1, followed by a 50-mg daily dose; 100-mg loading dose on day 1, followed by 70-mg daily dose; 70-mg daily dose; and 100-mg daily dose, with the population weight averages of 50 kg, 78 kg (population median), and 120 kg. The weight-based dosing regimens consisted of a 2-mg/kg loading dose followed by a 1-mg/kg daily dose; 2-mg/kg loading dose followed by a 1.25-mg/kg daily dose; 1.5-mg/kg loading dose followed by a 1.25-mg/kg daily dose; no loading dose and a 1-mg/kg daily dose; and no loading dose and a 1.5-mg/kg daily dose. A PTA of ≥90% was considered an optimal target.

Second, the PK/PD target AUC/MIC were analyzed, as these have been used in previous pharmacokinetic studies (9, 25, 33). An AUC/MIC target of 450 was used for Candida glabrata, 865 for Candida albicans, and 1,185 for Candida parapsilosis. These AUC/MIC targets are based on a preclinical murine study (24). PTAs were simulated for day 3 of therapy and for the MIC range of 0.01 to 1 mg/liter.

### Data availability.

Data are available upon request.

## Supplementary Material

Supplemental file 1
