# Supplementary material for: Caspofungin Weight-Based Dosing Supported by a Population Pharmacokinetic Model in Critically Ill Patients
Source: Antimicrob Agents Chemother. 2020 Aug 20;64(9):e00905-20. doi: 10.1128/AAC.00905-20 (PMC7449215; doi:10.1128/AAC.00905-20)

**Supplemental Table S1.** Tested models.

All covariate models were tested for a 2-compartment model with the same ranges as model 1 and 2.

|  | Characteristics (range of parameter limits) | AIC  (Aikake Information criterion) | BIC (Bayesian Information Criterion) | -2*Log- likelihood | Population fit (*r,* coefficient of determination value) | Individual fit (*r,* coefficient of determination value) | popBias (Population bias – mean weighted error of predicted-observed) | popper_RMSE (Population root mean squared error) |
| --- | --- | --- | --- | --- | --- | --- | --- | --- |
| 1 | K_e_ (0-5)  V (0.01-100)  No covariates | 462.5 | 472.6 | 456.4 | 0.543 | 0.564 | -0.547 | 36.79 |
| 2 | K_e_ (0-5)  V (0.01-100)  kcp (0-5)  kpc (0-5)  No covariates | 268.3 | 285.0 | 258.0 | 0.626 | 0.851 | -1.403 | 35.07 |
| 3 | Sex/V | 279.6 | 299.5 | 267.2 | 0.586 | 0.891 | -0.151 | 34.07 |
| 4 | Sex/Ke | 279.1 | 299.1 | 266.7 | 0.578 | 0.889 | 0.698 | 34.20 |
| 5 | SAPS/Ke | 408.5 | 425.1 | 398.2 | 0.491 | 0.608 | -6.965 | 86.57 |
| 6 | Weight(normalized)/V  Pop median: 78kg | 284.3 | 301.0 | 274.0 | 0.74 | 0.892 | 0.937 | 26.13 |
| 7 | Bilirubin/V | 452.6 | 469.3 | 442.3 | 0.276 | 0.401 | 0.773 | 87.86 |
| 8 | Bilirubin/Ke | 332.6 | 349.2 | 322.3 | 0.434 | 0.363 | -4.908 | 61.99 |
| 9 | Albumin/Ke | 313.4 | 330.1 | 303.3 | 0.548 | 0.68 | -7.915 | 77.46 |
| 10 | ASAT/Ke | 372.0 | 388.7 | 361.7 | 0.353 | 0.388 | -7.626 | 87.60 |
| 11 | ASAT/V | 712.3 | 728.9 | 702.0 | 0.215 | 0.255 | -2.129 | 72.42 |
| 12 | ALAT/V | 535.5 | 552.2 | 525.2 | 0.157 | 0.366 | -0.176 | 86.39 |
| 13 | Dialysis/Ke | 279.1 | 299.1 | 266.7 | 0.578 | 0.889 | 0.699 | 34.20 |
| 14 | Dialysis/V | 276.6 | 296.5 | 264.2 | 0.586 | 0.905 | -0.969 | 35.57 |
| 15 | Age/Ke | 376.0 | 392.6 | 365.7 | 0.489 | 0.538 | -7.291 | 84.13 |
| 16 | Age/V | 353.5 | 370.2 | 343.3 | 0.392 | 0.471 | -6.694 | 84.43 |
| 17 | Weight(normalized)/V  Dialysis/Ke | 278.2 | 298.1 | 265.8 | 0.751 | 0.908 | -0.635 | 26.68 |
| 18 | Weight(normalized)/V  Dialysis/V | 278.8 | 302.0 | 264.2 | 0.743 | 0.915 | 0.499 | 25.92 |
| 19 | Weight(normalized)/V  Dialysis/V  Dialysis/Ke | 280.2 | 306.6 | 263.5 | 0.751 | 0.809 | -2.052 | 33.64 |
| 20 | Weight(normalized)/V  K_e_ (0-0.4)  V0 (0.01-18)  kcp (0-2)  kpc (0-5)  Lambda = 1 | 320.8 | 337.4 | 310.5 | 0.751 | 0.959 | 0.346 | 25.51 |
| 21 | Weight(normalized)/V  K_e_ (0-0.4)  V0 (0.01-18)  kcp (0-2)  kpc (0-5)  Lambda = 0.2 | 319.9 | 336.5 | 309.6 | 0.752 | 0.958 | 0.436 | 25.57 |
| 22 | Weight(normalized)/V  K_e_ (0-0.4)  V0 (0.01-18)  kcp (0-2)  kpc (0-5)  Gamma = 5 | 258.6 | 275.2 | 248.3 | 0.751 | 0.963 | 0.861 | 25.71 |
| 23 | Weight(normalized)/V  K_e_ (0-0.4)  V0 (0.01-18)  kcp (0-2)  kpc (0-5)  Gamma = 1 | 247.8 | 258.1 | 274.7 | 0.752 | 0.958 | 1.275 | 26.51 |
| 24 | **Final model:**  **Optimized margins**  Weight(normalized)/V  K_e_ (0-0.4)  V0 (0.01-18)  kcp (0-2)  kpc (0-5)  Gamma = 2 | **258.6** | **275.2** | **248.3** | **0.752** | **0.96** | **0.861** | **25.71** |

**Supplemental Figure S1.** External validation with an independent dataset (Muilwijk et al 2014).


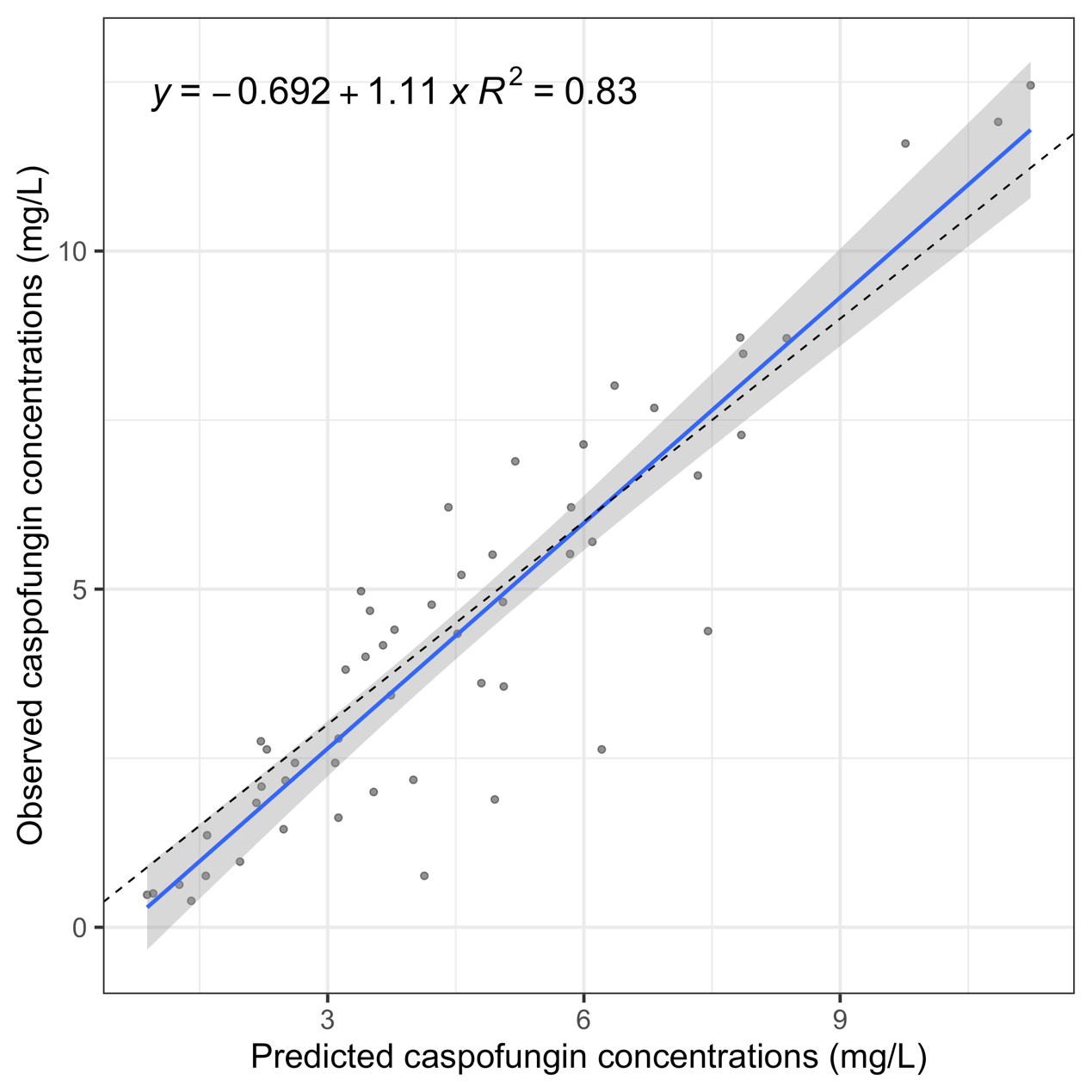


**Supplemental Figure S2.** Normalised prediction distribution error (NPDE) plots


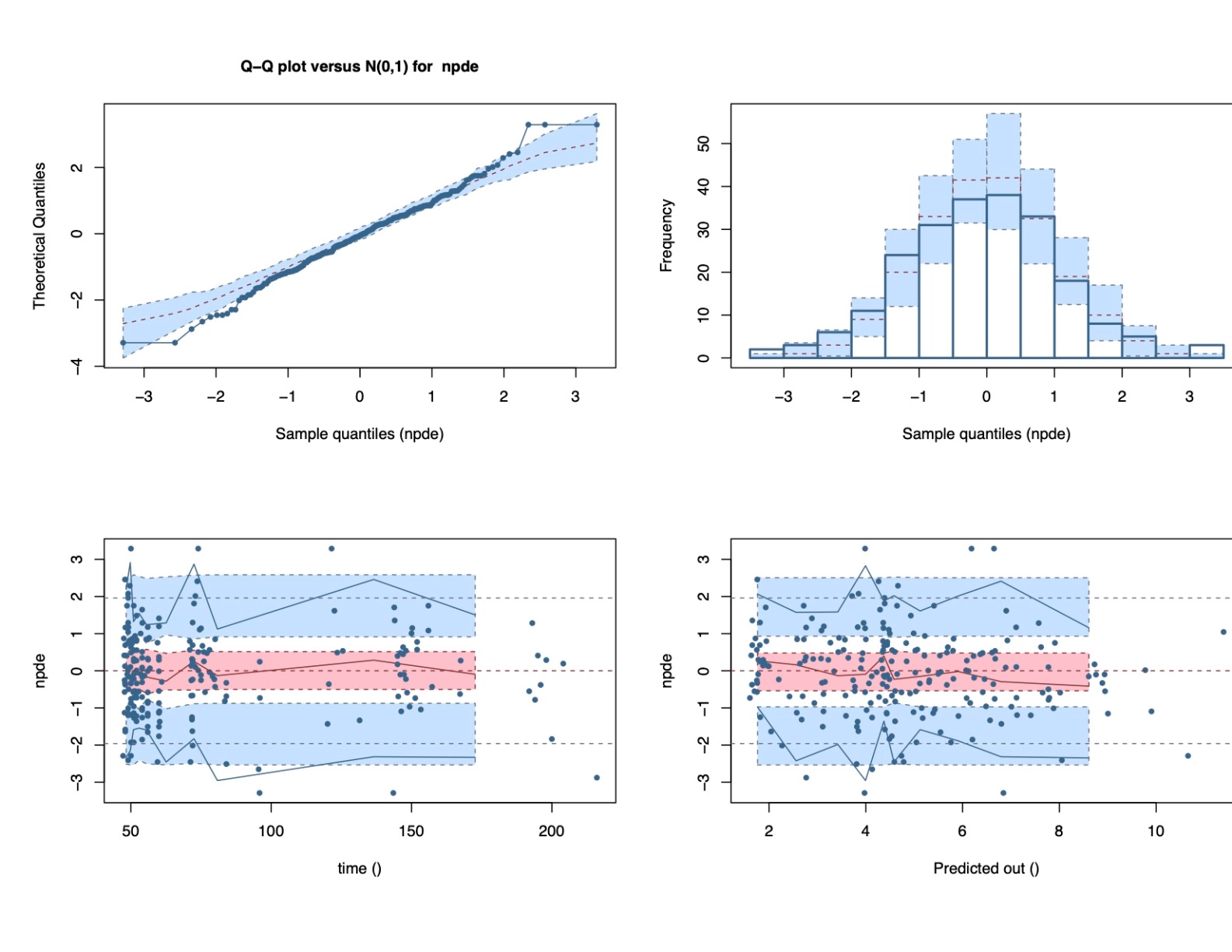

Supplement: Supplemental file 1 [file AAC.00905-20-s0001.docx]
